# Community Responses during Early Phase of COVID-19 Epidemic, Hong Kong

**DOI:** 10.3201/eid2607.200500

**Published:** 2020-07

**Authors:** Kin On Kwok, Kin Kit Li, Henry Ho Hin Chan, Yuan Yuan Yi, Arthur Tang, Wan In Wei, Samuel Yeung Shan Wong

**Affiliations:** JC School of Public Health and Primary Care, The Chinese University of Hong Kong, Hong Kong, China (K.O. Kwok, H.H.H. Chan, Y.Y. Yi, W.I. Wei, S.Y.S. Wong);; Stanley Ho Centre for Emerging Infectious Diseases, The Chinese University of Hong Kong, Hong Kong (K.O. Kwok);; Shenzhen Research Institute of The Chinese University of Hong Kong, Shenzhen, China (K.O. Kwok);; City University of Hong Kong College of Liberal Arts and Social Sciences, Hong Kong (K.K. Li);; Sungkyunkwan University College of Software, Seoul, South Korea (A. Tang)

**Keywords:** COVID-19, 2019 novel coronavirus disease, coronavirus disease, SARS-CoV-2, severe acute respiratory syndrome coronavirus 2, viruses, respiratory infections, zoonoses, Hong Kong

## Abstract

During the early phase of the coronavirus disease epidemic in Hong Kong, 1,715 survey respondents reported high levels of perceived risk, mild anxiety, and adoption of personal-hygiene, travel-avoidance, and social-distancing measures. Widely adopted individual precautionary measures, coupled with early government actions, might slow transmission early in the outbreak.

Hong Kong was relatively successful in mitigating transmission early in the outbreak of coronavirus disease (COVID-19). Confirmed cases were first reported in the city of Wuhan, China, in December 2019 (*1*). Situated at the southern tip of China, Hong Kong was at risk for importing COVID-19, given its shared border and high infrastructural and social connectivity with China. In 2019, >236 million passengers crossed the border between China and Hong Kong by land (*2*). Hong Kong is also vulnerable to virus transmission owing to its high population density and heavy reliance on public transportation. Despite these risks, as of March 20, 2020, transmission control efforts in Hong Kong, as reflected in the numbers of confirmed cases and deaths (256 cases, 4 deaths) (*3*), had been relatively successful compared with nearby countries and regions, including mainland China (80,967 cases, 3,248 deaths), South Korea (8,652 cases, 94 deaths), and Japan (950 cases, 33 deaths, in addition to the 712 cases from a cruise ship) (*4*).

Health officials in Hong Kong have enacted multipronged interventions to slow disease spread (*5*). Adopted strategies include border screening (measuring body temperature, imposing a health declaration form system, imposing a 14-day mandatory quarantine period on persons entering Hong Kong from mainland China; parts of Korea, Japan, France, Germany, and Spain; and all of Italy and Iran), social distancing (shutting down the border, reducing cross-border commuting services, delaying the resumption of classes in schools, arranging telework for civil servants, and suspending of public services), and extending the Enhanced Laboratory Surveillance Program to adult patients with fever and mild respiratory symptoms at emergency departments or general outpatient clinics in the public sector.

The behaviors of the public are important for outbreak management, particularly during the early phase when no treatment or vaccination is available and nonpharmaceutical interventions are the only options. The efficacy of nonpharmaceutical interventions depends on persons’ degree of engagement and compliance in precautionary behaviors, such as face-mask wearing, hand hygiene, and self-isolation. Willingness to engage in precautionary behaviors voluntarily depends on risk perception toward the current health threat. In fact, risk perception is a main theme in common health behavior theories (*6*,*7*). In addition, with advanced information technology in recent years comes the uncertainty of how risk perception is shaped by various information sources. Hong Kong’s experience with outbreaks of novel pathogens (e.g., 2003 severe acute respiratory syndrome [SARS] and 2009 pandemic influenza) also provides a reference point to evaluate the risk perceptions of COVID-19. In comparison, Hong Kong was more affected by SARS than COVID-19 thus far. In 2003, a total of 1,755 persons in Hong Kong contracted SARS, resulting in 299 deaths (*8*).

In light of the importance of persons’ behavior in mitigating transmission and the goal of informing policy formation in a timely manner, we examined risk perceptions and behavioral responses of the general community during the early phase of the COVID-19 epidemic in Hong Kong. Considering the rapid development of the epidemic during the survey period and the potential variability in the adoption of preventive measures among persons, we also examined the temporal changes in anxiety levels, the factors associated with adoption of preventive measures, and sources of information about the epidemic.

## The Study

**Figure 1:**
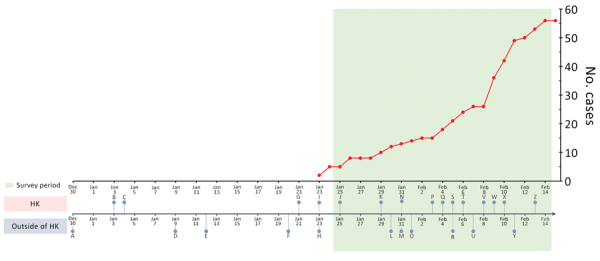
Chronology of major events during the early phase of the coronavirus disease epidemic and laboratory-confirmed cases in Hong Kong, December 30, 2019–February 14, 2020. A, unexplained pneumonia reported in Wuhan, China; B, HK begins temperature screenings at border checkpoints for travelers from Wuhan; C, HK launches preparedness and response plan for novel infectious disease of public health significance, serious response level; D, first death reported in Wuhan; E, World Health Organization (WHO) names disease 2019-nCoV acute respiratory disease and the virus 2019-nCoV (refer to Y for subsequent renaming); F, China confirms human-to-human transmission; G, HK introduces health declaration form system on inbound travelers by air from Wuhan; H, WHO declines to declare COVID-19 a public health emergency of international concern; I, first first confirmed COVID-19 case in HK, halt of sale of high-speed rail tickets to and from Wuhan; J, HK activates emergency response level; K, HK closes public leisure and cultural facilities until further notice; L, WHO declares COVID-19 a public health emergency of international concern; M, United States declares COVID-19 a public health emergency, imposes entry restriction; N, HK imposes 4-week school suspension, 1-week extension for home-office arrangement for civil servants; O, first COVID-19 death outside China in the Philippines; P, HK medical workers strike to call for border shutdown; Q, first COVID-19 death in HK, closure of 4 more border control points; R, 46 foreign airlines cancelled flights to mainland China; S, HK implements further port hygiene measures; T, HK offers home-office arrangement for civil servants until February 16; U, first death of a doctor in China (Wuhan); V, HK begins mandatory 14-day quarantine on persons entering from China; W, HK reports COVID-19 cluster involving 9 people in a gathering on January 26; X, HK reports COVID-19 cluster involving 5 residents (2 families) in the same building; Y, WHO and ICTV rename disease COVID-19 and virus SARS-CoV-2; Z, HK extends home-office arrangement for civil servants until February 23, school suspension until March 16. HK, Hong Kong.

District councilors distributed an online survey including measures of preventive behaviors, general anxiety, risk perceptions, and information exposure to the residents of Hong Kong by within 36 hours after detection of the first confirmed case of COVID-19 in Hong Kong (Appendix). The survey was conducted for 3 weeks. We compiled a chronology of major events related to COVID-19 both inside and outside Hong Kong and the number of confirmed cases in Hong Kong before and during the period covered by the survey (Figure 1). 

Analysis of 1,715 respondents’ data indicated high levels of perceived susceptibility to (89%) and severity of (97%) COVID-19 (Table 1). However, the general anxiety level, measured by the Hospital Anxiety and Depression Scale (*9*), was mild (9.01 out of 21). Most respondents (>98%) had their daily routines disrupted and were alert to COVID-19. The most trusted information sources were doctors (84%) and radio broadcasts (57%), but they were not the sources by which respondents typically received their information (doctors 5%, broadcast 34%). 

**Table 1:** Risk perception of the community toward COVID-19 during the early phase of the COVID-19 epidemic in Hong Kong*

Characteristic	No. (%) respondents				
	Level 1	Level 2	Level 3	Level 4	Level 5
Perceived susceptibility (assuming no preventive measure)					
How likely you will be infected†	776 (45)	751 (44)	160 (9)	23 (1)	5 (0)
How likely your families will be infected†	924 (54)	660 (38)	113 (7)	14 (1)	4 (0)
Perceived severity					
Seriousness of symptoms caused by SARS-CoV-2‡	1102 (64)	569 (33)	33 (2)	7 (0)	4 (0)
Chance of having COVID-19 cured§	190 (11)	552 (32)	708 (41)	239 (14)	26 (2)
Chance of survival if infected with COVID-19§	136 (8)	476 (28)	788 (46)	290 (17)	25 (1)

*COVID-19, coronavirus disease; SARS-CoV-2, severe acute respiratory syndrome coronavirus 2. †Level 1, very likely; level 2, likely; level 3, neutral; level 4, unlikely; level 5, very unlikely ‡Level 1, very serious; level 2, serious; level 3, neutral; level 4, not serious; level 5, not serious at all §Level 1, very low; level 2, low; level 3, neutral; level 4, high; level 5, very high

**Figure 2:**
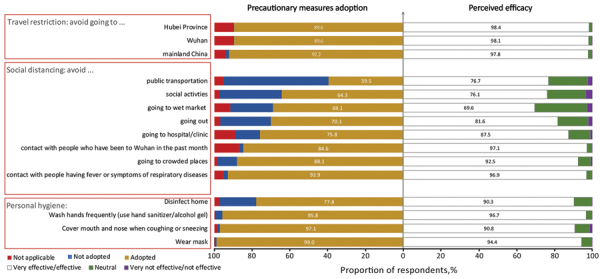
Perceived efficacy and actual adoption of precautionary measures to prevent transmission of severe acute respiratory syndrome coronavirus 2 and avoid contracting coronavirus disease, Hong Kong.

Among preventive measures and their perceived efficacy, enhanced personal hygiene (from 78% of respondents disinfecting their homes to 99% wearing facemasks) and travel avoidance (from 90% avoiding Hubei Province, China, to 92% avoiding mainland China altogether) were frequently adopted and were considered effective (>90%) (Figure 2). The adoption of social-distancing measures was moderate to high (from 40% respondents avoiding public transportation to 93% avoiding contact with persons with respiratory disease symptoms). Higher levels of adoption of social-distancing measures were associated with being female, living in the New Territories (1 of the 3 geographic regions in Hong Kong that shares the border with mainland China), perceiving oneself as having a good understanding of COVID-19, and being more anxious (Table 2). 

**Table 2:** Factors associated with greater adoption of social-distancing interventions during the early phase of the COVID-19 epidemic in Hong Kong*

Characteristic	aOR (95% CI)	p value†
Sex		
M	Referent	
F	1.31 (1.06–1.63)	0.01
Age group, y		
18–24	Referent	
25–34	1.26 (0.97–1.63)	0.08
35–44	1.17 (0.88–1.56)	0.28
45–54	1.34 (0.94–1.92)	0.11
>55	0.93 (0.61–1.41)	0.74
District of residence		
Hong Kong Island	Referent	
Kowloon East	0.96 (0.68–1.36)	0.83
Kowloon West	0.95 (0.62–1.46)	0.82
New Territories East	1.57 (1.18–2.11)	0.00
New Territories West	1.37 (1.02–1.85)	0.04
Left Hong Kong in the previous month		
No	Referent	
Yes	0.72 (0.57–0.91)	0.01
Made regular visits to mainland China		
No	Referent	
Yes	0.48 (0.24–0.91)	0.03
Perceived understanding about COVID-19		
Not well or not well at all	Referent	
Neutral	1.07 (0.76–1.51)	0.70
Well or very well	1.80 (1.27–2.56)	0.00
Presence of chronic diseases		
No	Referent	
Yes	0.77 (0.55–1.06)	0.11
Anxiety level		
Normal	Referent	
Mild	1.38 (1.08–1.76)	0.01
Moderate or severe	1.71 (1.34–2.17)	0.00

*aOR, adjusted odds ratio; COVID-19, coronavirus disease. †By 2-tailed *t*-test.

## Conclusions

The relative success in transmission control in Hong Kong could be attributed to the widely adopted precautionary behaviors of the public, together with early government interventions (e.g., border control and compulsory quarantine for those from affected regions). Unlike in many other countries, visitors from mainland China have never been fully banned from entering Hong Kong. The citizens of Hong Kong assumed responsibility for infection control on their own and became very attentive to personal preventive measures. Our findings showed that nearly all respondents adopted enhanced personal hygiene (e.g., wearing facemasks) and travel avoidance. The experience in outbreak management during the 2003 SARS epidemic might also have contributed to these swift and strong psychological and behavioral responses. Metaphorically, these responses resembled a secondary immune response, which is fast and strong during re-exposure to the same pathogen.

The case of Hong Kong demonstrates the extent to which voluntary preventive measures by persons might be required for slowing transmission (e.g., >78% adoption of enhanced personal-hygiene measures, >90% adoption of travel-avoidance, and 39%–93% adoption of social-distancing). Being in agreement with the findings of Anderson et al. (*10*), we hope that these behavioral standards are useful in promoting person-level preventive measures for countries in the early phase of the COVID-19 outbreak, especially when border-control measures are not viable. This high level of civil engagement toward disease control also enables most business to continue as usual, which reduces the economic toll from strict quarantine measures.

In addition, we consider the increased anxiety levels reported as a double-edged sword. On one hand, anxiety can motivate precautionary measures. On the other hand, it might adversely affect school, work, or family life. Besides providing accurate information about the epidemic, public health institutions (e.g., Hong Kong Department of Health) also should promote a healthy lifestyle and psychological well-being. Further discussion of the interpretation of some specific findings, including assessing the sustainability of the preventive measures, the general anxiety level of the public in different outbreaks, the effective communication channels for COVID-19 information, and the drivers of social-distancing behaviors are provided (Appendix).

In conclusion, we identified high levels of risk perception regarding COVID-19 in the community in Hong Kong. Most respondents were alert to the disease progression of COVID-19 and adopted self-protective measures. Our findings contribute to the body of research examining the psychobehavioral responses of the public, in addition to the already widely studied biologic and mechanistic aspects of COVID-19, during the early phase of the current COVID-19 epidemic. The timely psychologic and behavioral assessment of the community can inform subsequent intervention and risk-communication strategies as the epidemic progresses.

Appendix Additional information about community responses during early phase of COVID-19 epidemic, Hong Kong.
