# Supplementary material for: Community Responses during Early Phase of COVID-19 Epidemic, Hong Kong
Source: Emerg Infect Dis. 2020 Jul;26(7):1575–9. doi: 10.3201/eid2607.200500 (PMC7323558; doi:10.3201/eid2607.200500)
Supplement: Appendix — Additional information about community responses during early phase of COVID-19 epidemic, Hong Kong. [file 20-0500-Techapp-s1.pdf]

Article DOI: https://doi.org/10.3201/eid2607.200500

Community Responses during Early Phase of COVID-19 Epidemic, Hong Kong

Appendix

Methods

Subject Recruitment

A cross-sectional online survey was conducted within 36 hours after the first confirmed Coronavirus Disease 2019 (COVID-19) case was reported in Hong Kong. To ensure good coverage of the general community in Hong Kong, chairpersons and vice-chairpersons of all eighteen district councils and all individual councilors of the 452 District Council Constituency Areas (DCCAs) were approached by electronic mails and their contact numbers listed in the District Council Web sites (https://www.districtcouncils.gov.hk/index.html) for survey dissemination. District councilors were invited to share our survey link and promotion messages on their webpages, social media platforms or any channels which they usually use to convey information to their targeted residents, but in general there was no restriction on their dissemination. Individuals who were aged 18 or above, understood Chinese and lived (on average) over 5 days per week in Hong Kong in the last month were eligible to participate. Respondents were compensated with a HKD10 cash coupon if they indicated willingness for receipt. To avoid duplicated responses from the same respondent, the survey could only be taken once from the same electronic device. To start this survey, respondents were asked to indicate their willingness for participation by answering this first question "Are you willing to participate?." Only those who answered "Yes" could go on with the survey.

Respondent Characteristics

Respondents were asked about their demographics (including sex, age, living district, education attainment, household income), self-perceived health status, travel history in the past month, occurrence of respiratory symptoms in the past fourteen days. The scales and response sets of these measures are self-explanatory in the respective result tables. Respondents were also

asked about anxiety level using the Chinese-Cantonese version of the Hospital, Anxiety and Depression scale - Anxiety (HADS-A) (0–7 = Normal or no anxiety; 8–10 = mild anxiety; 11–14 = moderate anxiety; 15–21 = severe anxiety) (*1*). Although HADS-A is intended for screening clinically significant anxiety symptoms in clinical populations, many studies have showed that it is valid for community populations (*2*,*3*), including employees (*4*), general population aged 65–80 years in Sweden (*5*), an Italian community sample aged 18–85 years (*6*). As a complementary measure, the state anxiety level of a subset of respondents was assessed on a four-point scales (1 = almost never; 4 = almost always) with the validated Chinese version of State-Trait Anxiety Inventory (STAI) (*7*) adapted from the original STAI (*8*). State anxiety from a 10-item STAI was used when comparing to past studies wherever applicable.

Risk Perception

Risk perception toward COVID-19 was measured by two very relevant psychological dimensions (*9*): (i) perceived susceptibility, and (ii) perceived severity. The first dimension was proxied by how likely one considered oneself (his/her families) would be infected with COVID-19 if no preventive measure was taken. The second dimension was proxied by how one rated the seriousness of symptoms caused by COVID-19, their perceived chance of having COVID-19 cured and that of survival if infected with COVID-19. The items are shown in Table 1 (https://wwwnc.cdc.gov/EID/article/26/7/20-0500-T1.htm). Subjects were also asked to rate the relative severity of COVID-19 compared with common non-communicable diseases (NCDs) and previous outbreaks by novel pathogens in Hong Kong. Responses were captured with a five-point Likert scale.

Information Exposure

Respondents were asked about the sources from which they obtained information about COVID-19, and how much they trust those sources. They were also asked about the types of information that they wanted to receive.

Preventive Measures

Respondents were asked whether they performed precautionary measures and what their perceived efficacy of those measures are. Three types of precautionary measures were considered: hygienic practices, social distancing and travel avoidance.

Ethics Consideration

This study has been approved by the Survey and Behavioral Research Ethics Committee of The Chinese University of Hong Kong.

Statistical Analysis

Frequency and proportions of responses were tabulated. Demographics of respondents were compared to the 2016 population by-census in Hong Kong with Cohen's w effect size (small: 0.1; medium: 0.3; large: 0.5) (*10*). Regression models were used to test for temporal change in anxiety level and to identify factors associated with greater adoption of social-distancing preventive measures (proxied by adopting five or more social-distancing precautionary measures). For temporal change in anxiety level, the HADS-A score was considered the outcome with the survey date being the exposure. Adjusted odds ratio (aOR) and 95% confidence interval (CI) were estimated. Candidate variables included: demographics of respondents, self-perceived health status, travel history and anxiety level. A statistical significance of 0.05 was specified. Analysis was performed in R.

Results

The survey was conducted from 24 January 2020 to 13 February 2020 (Figure 1, https://wwwnc.cdc.gov/EID/article/26/7/20-0500-F1.htm). Our survey period covers important clinical incidences, including first local death case and first overseas death case (Philippines), and social incidences, including healthcare workers on strike to call for entire border shutdown. It was also amid of the start-up of large-scale social-distancing interventions, including halt of sales of high-speed rail tickets to and from Wuhan, closure of public cultural and leisure facilities and deferral of school resumption. Meanwhile, alongside the launch of this survey was the escalating official threat tone on COVID-19: The World Health Organization (WHO) declared the COVID-19 epidemic as a public health emergency of international concern, with Hong Kong activated the emergency response level.

Respondent Characteristics

There were initially 2478 clicks of the survey link which fulfill the inclusion criteria, with 6%–31% missingness on demographics variables. Therefore, for a complete-case analysis, 1715 responses were analyzed. Appendix Table 1 shows the respondent characteristics. Many of the

respondents are female (69%; 1176/1715), of young age (18–44 years) (80%; 1380/1715), working population (68%; 1168/1715). The study sample is moderately comparable to the population in terms of living district and sex (effect size = 0.27). Appendix Table 2 shows the background health conditions and travel history of respondents. The majority perceived their health status as good or very good (78%; 1331/1715), a quarter of them experienced respiratory symptoms in the past 14 days (25%; 423/1715) and traveled outside Hong Kong in the previous month (24%; 408/1715). Among the 408 respondents who were abroad, at least 24% of them went to the Mainland China excluding Macau.

Risk Perception

Table 1 (https://wwwnc.cdc.gov/EID/article/26/7/20-0500-T1.htm) shows the perceived susceptibility and perceived severity toward COVID-19 among respondents. Most respondents regarded themselves as likely to be infected with COVID-19 (very likely/likely: 89%), and most considered the symptoms of COVID-19 (if infected) as serious (very serious/serious: 97%). Less than a quarter of the respondents thought that it was likely to have COVID-19 cured (if infected) (15%), and only 18% thought that it was likely to survive through COVID-19. When referencing to existing diseases (Appendix Table 3), almost all respondents (>98%) consider equivalent disease severity between COVID-19 and SARS. This magnitude was similar to other deadly NCDs (85%–96%), but much higher than the annual seasonal influenza (66%).

Most respondents were worried about COVID-19 (97%; 1667/1715), and they claimed that their daily routines were slightly (42%; 727/1715) or greatly (56%; 955/1715) disrupted. The average HADS-A score is 9.01 out of 21 (standard deviation [SD]:4.23); while the average score of state anxiety by the full-version STAI, from 804 complete responses, is 2.66 (SD: 0.58). A significantly increasing time trend in HADS-A score is identified ($p < 0.05$) (Appendix Figure 1).

Information Exposure

Nearly all respondents were continuously alert to the disease progression of COVID-19 (99.5%; 1707/1715) and actively searched for related information (83%; 1431/1715). Appendix Table 4 lists the types of COVID-19 information wanted by the 1639 (96%) respondents who indicated such need. Information which respondents were most interested were: distribution of

cases (92%), number of infected individuals (91%), infection control interventions undertaken by local officials (88%), and preventive measures (87%).

Appendix Figure 2 shows the sources from which respondents obtained information about COVID-19, and how well the information sources were trusted. The most trusted sources were doctors (84%; very reliable/reliable: 1449/1715), but only 5% (87/1715) respondents could obtain information from them. The next two most trusted sources were broadcast (57%) and newspaper (54%), but they were used by less than 40% of the respondents. On the other hand, the two most common information sources were social platforms (94%; 1608/1715) and Web sites (regardless of official or unofficial) (90%; 1539/1715), but they were rated as reliable or very reliable by only 26% and 16%–23% of the respondents respectively. Only 16% (269/1715) of respondents found information from official Web sites reliable or very reliable.

Preventive Measures

Figure 2 ((https://wwwnc.cdc.gov/EID/article/26/7/20-0500-F2.htm)shows the adoption of precautionary measures by respondents and their perceived efficacy. Enhanced personal hygiene practices (including wearing masks, cleaning hands and better coughing and sneezing etiquette) and avoid traveling to Mainland China were adopted by most respondents (>89%), and these practices were considered very effective or effective (>90%). For social-distancing measures, although they were considered useful in preventing COVID-19 (very effective/effective: ≥70%), their actual adoption was moderate-to-high (range 39%–93%).

Table 2 (https://wwwnc.cdc.gov/EID/article/26/7/20-0500-T2.htm) shows the regression analysis results for greater adoption (five or more) of social-distancing interventions during the early phase of this COVID-19 epidemic. Being female (aOR 1.31; 95% CI 1.06–1.63), living in the NT (aOR 1.37–1.57), perceived as having good understanding of COVID-19 (aOR 1.80; 95% CI 1.27–2.56), being more anxious (aOR 1.38–1.71) were positively associated with greater adoption.

Discussion

This study provides timely assessment of the risk perception, information exposure and adoption of precautionary measures during the initial phase of the COVID-19 epidemic in Hong Kong. Despite disease uncertainty (including transmissibility, route of transmission and

pathogenicity) at the early stage, individuals in the community had high perceived risk toward COVID-19 at large, viz: high perceived susceptibility and high perceived severity. A slightly increasing general anxiety level was observed over the 3-week study period. Enhanced personal hygiene and travel avoidance were adopted by nearly all respondents, higher propensity of adopting greater degree of social-distancing measures were associated with being female, living in the New Territories, perceived as having good understanding of COVID-19 well, work status except students and being more anxious. Our results have several immediate and significant public health implications.

First, our results provide the baseline psychological and behavioral responses of the community against which current infection control strategies fit in. With the high perceived risk and large proportion of individuals adopting preventive measures in the community at the beginning, during which the accumulated number of local cases is 130 (almost 7 weeks since the first case) with a significant initial portion of them being imported cases (*11*), we have an edge to block local transmission. This suggests that efforts to curb imported cases were efficient at the early phase of this outbreak. Following the enactment of a 14-day quarantine period for individuals entering Hong Kong from the Mainland China, Italy, Iran, and other regions with outbreaks, and the emergence of clustered local cases, the next important strategy on the agenda is to stabilize the supply of preventive materials, such as masks, so that the blockage of local transmission chain can be sustained. Besides sustainable supply, how much longer the public can maintain this high adoption of preventive measures without seeing the light at the end of tunnel has become a concern. Follow-up studies on the sustainability of such behaviors are needed.

Second, our results reveal the risk perception in the community, which is an important piece of information to enhance epidemic control (*12*). Although the epicenter of the COVID-19 epidemic is Wuhan, the perceived risk of the community in Hong Kong was high. For emotional status, the HADS-A score in our survey (9.01 out of 21) suggests that the community had mild anxiety. The community was more anxious about the current COVID-19 epidemic (mean of the 10-item state anxiety from STAI = 2.57; SD = 0.62) than the 2003 SARS outbreak (mean of the 10-item state anxiety from STAI = 2.24; SD = 0.58) (p-value from two-sample t-test <0.05) (*13*). The significant time trend associated with HADS-A (Appendix Figure 1) suggests that the community became more and more anxious as new cases and new incidences came up (Figure 1).

Third, our results suggest an alternative strategy for better risk communication. The large proportion of respondents were alert to COVID-19 (99.5%) or actively searching for related information (83%) highlighted the role of social media in shaping risk perception and epidemic-related emotion. It is particularly important amid of much disease uncertainty as mass scares can be triggered easily. Considering the high level of trust given by respondents to doctors and the low level of trust to the two most frequently used information sources, social platform and Web sites, health officials can collaborate frequently with associations of medical doctors, and invite them to help propagating official information in more sociable channels. This strategy is deemed more acceptable by the community than relying solely on the official channel, given only 16% of respondents rates official Web sites as reliable or very reliable. Our results also shortlisted information preferred by the community among an upsurge of disease-related information during the early stage (Appendix Table 4).

Fourth, our results pinpoint the drivers for greater level of adoption of social-distancing precautionary measures. In line with literatures that being female and an elevated anxiety level prompted compliance of precautionary measures (*13*,*14*), we also identified similar association in this survey (Table 2, (https://wwwnc.cdc.gov/EID/article/26/7/20-0500-T2.htm)). Interestingly, specific to this COVID-19 epidemic, residents in the New Territories were more likely to comply with social-distancing precautionary measures than their counterparts in other areas of Hong Kong. Separating Hong Kong and the Guangdong Province are two busiest custom borders, Lo Wu and Lok Ma Chau, such that the residents in the New Territories may consider themselves at greater risk of infection. Those who claimed they understood COVID-19 were more likely to adopt preventive measures, suggesting mass promotion of knowledge about COVID-19 in the community can boost uptake of precautionary measures. On the other hand, the less propensity to adopt precautionary measures among individuals who left Hong Kong in the previous month or who regularly visited China. Some of these visits might be work- or family-related. Social interactions became difficult to avoid during travels such as the use of public transportation, going out, and going to crowded places (as mentioned above, the borders are always crowded except with border control). These findings reinforce the need for border screening and for promoting social hygiene amid of epidemic times.

Fifth, this local study has profound implication to overseas countries undergoing the initial phase of the COVID-19 epidemic. The WHO European region has been accumulating

COVID-19 cases, but in only 4 days (22–25 February 2020), the number of laboratory-confirmed cases in Italy has risen from 9 to 229 (*15–18*). Recently on 24 February 2020, the Ministry of Health announced the first COVID-19 case in Iraq. The presence of initial cases, aligning with the human-to-human (*19*) and asymptomatic (*20*) transmission, suggest that many countries may experience the initial phase of the COVID-19 epidemic soon. Results of this survey serve as a reference for overseas health officials to better prepare their containment strategies and handle the potential mass scares in their community.

Strengths and Limitations

This study has two strengths. First, it started within 36 hours after the detection of first local cases. This early start enables timely assessment of the community responses such that there is sufficient gap period to inform intervention policies. Second, our recruitment method, online survey via dissemination by DCCA councilors, is the first of its kinds to capture responses during public holidays while maintaining good geographic representation. The COVID-19 epidemic was amid of the Chinese New Year holidays and a series of large-scale social-distancing interventions enacted by Hong Kong government, particularly the home-office arrangement for employees. Therefore, the conventional random digit dialing approach adopted in the past local outbreaks (*13,21,22*) was not possible. And the involvement of all 452 DCCA councilors allows a thorough representation of every district in Hong Kong in the absence of a universal email database.

This study has two limitations. First, with an online approach, responses of those without internet access, particularly the oldest age group (55 years or above), were under-represented. Despite this, online surveys were the only feasible means of data collection during outbreak times. Second, this survey was conducted during the early phase that temporal variations of responses are not captured as the epidemic progresses. However, contact information were collected from this study cohort and follow-up surveys will be carried out as the disease progresses.

References

1. Leung CM, Wing YK, Kwong PK, Lo A, Shum K. Validation of the Chinese-Cantonese version of the hospital anxiety and depression scale and comparison with the Hamilton Rating Scale of

Depression. Acta Psychiatr Scand. 1999;100:456–61. PubMed https://doi.org/10.1111/j.1600-0447.1999.tb10897.x

2. Bjelland I, Dahl AA, Haug TT, Neckelmann D. The validity of the Hospital Anxiety and Depression Scale. An updated literature review. J Psychosom Res. 2002;52:69–77. PubMed https://doi.org/10.1016/S0022-3999(01)00296-3

3. Snaith RP. The Hospital Anxiety And Depression Scale. Health Qual Life Outcomes. 2003;1:29. PubMed https://doi.org/10.1186/1477-7525-1-29

4. Bocéréan C, Dupret E. A validation study of the Hospital Anxiety and Depression Scale (HADS) in a large sample of French employees. BMC Psychiatry. 2014;14:354. PubMed https://doi.org/10.1186/s12888-014-0354-0

5. Djukanovic I, Carlsson J, Årestedt K. Is the Hospital Anxiety and Depression Scale (HADS) a valid measure in a general population 65-80 years old? A psychometric evaluation study. Health Qual Life Outcomes. 2017;15:193. PubMed https://doi.org/10.1186/s12955-017-0759-9

6. Iani L, Lauriola M, Costantini M. A confirmatory bifactor analysis of the Hospital Anxiety and Depression Scale in an Italian community sample. Health Qual Life Outcomes. 2014;12:84. PubMed https://doi.org/10.1186/1477-7525-12-84

7. Shek DT. The Chinese version of the State-Trait Anxiety Inventory: its relationship to different measures of psychological well-being. J Clin Psychol. 1993;49:349–58. PubMed https://doi.org/10.1002/1097-4679(199305)49:3<349::AID-JCLP2270490308>3.0.CO;2-J

8. Spielberger CD, Gorsuch RL, Lushene R, Vagg PR, Jacobs GA. Manual for the State-Trait Anxiety Inventory. Palo Alto, California: Consulting Psychologists Press; 1983.

9. El-Toukhy S. Parsing susceptibility and severity dimensions of health risk perceptions. J Health Commun. 2015;20:499–511. PubMed https://doi.org/10.1080/10810730.2014.989342

10. Cohen J. Statistical power analysis for the behavioral sciences. 2nd ed. Hillsdale, N.J.: L. Erlbaum Associates; 1988.

11. Hong Kong Centre for Health Protection. Latest situation of cases of COVID-19. 2020 [cited 2020 Mar 21]. https://www.chp.gov.hk/files/pdf/local_situation_covid19_en.pdf

12. Herrera-Diestra JL, Meyers LA. Local risk perception enhances epidemic control. PLoS One. 2019;14:e0225576. PubMed https://doi.org/10.1371/journal.pone.0225576

13. Leung GM, Lam TH, Ho LM, Ho SY, Chan BH, Wong IO, et al. The impact of community psychological responses on outbreak control for severe acute respiratory syndrome in Hong Kong. J Epidemiol Community Health. 2003;57:857–63. PubMed https://doi.org/10.1136/jech.57.11.857

14. Bults M, Beaujean DJ, de Zwart O, Kok G, van Empelen P, van Steenbergen JE, et al. Perceived risk, anxiety, and behavioural responses of the general public during the early phase of the Influenza A (H1N1) pandemic in the Netherlands: results of three consecutive online surveys. BMC Public Health. 2011;11:2. PubMed https://doi.org/10.1186/1471-2458-11-2

15. World Health Organization. Coronavirus disease (COVID-2019) Situation Report - 33. 2020 [cited 2020 Mar 25]. https://www.who.int/docs/default-source/coronaviruse/situation-reports/20200222-sitrep-33-covid-19.pdf?sfvrsn=c9585c8f_4

16. World Health Organization. Coronavirus disease (COVID-2019) Situation Report - 34. 2020 [cited 2020 Mar 25]. https://www.who.int/docs/default-source/coronaviruse/situation-reports/20200223-sitrep-34-covid-19.pdf?sfvrsn=44ff8fd3_2

17. World Health Organization. Coronavirus disease (COVID-2019) Situation Report - 35. 2020 [cited 2020 Mar 25. https://www.who.int/docs/default-source/coronaviruse/situation-reports/20200224-sitrep-35-covid-19.pdf?sfvrsn=1ac4218d_2

18. World Health Organization. Coronavirus disease (COVID-2019) Situation Report - 36. 2020 [cited 2020 Mar 25]. https://www.who.int/docs/default-source/coronaviruse/situation-reports/20200225-sitrep-36-covid-19.pdf?sfvrsn=2791b4e0_2

19. Paules CI, Marston HD, Fauci AS. Coronavirus Infections-More Than Just the Common Cold. JAMA. 2020;323:707; Epub ahead of print. PubMed https://doi.org/10.1001/jama.2020.0757

20. Bai Y, Yao L, Wei T, Tian F, Jin DY, Chen L, et al. Presumed Asymptomatic Carrier Transmission of COVID-19. JAMA. 2020; Epub ahead of print. PubMed https://doi.org/10.1001/jama.2020.2565

21. Lau JT, Yang X, Tsui H, Kim JH. Monitoring community responses to the SARS epidemic in Hong Kong: from day 10 to day 62. J Epidemiol Community Health. 2003;57:864–70. PubMed https://doi.org/10.1136/jech.57.11.864

22. Cowling BJ, Ng DM, Ip DK, Liao Q, Lam WW, Wu JT, et al. Community psychological and behavioral responses through the first wave of the 2009 influenza A(H1N1) pandemic in Hong Kong. J Infect Dis. 2010;202:867–76. PubMed https://doi.org/10.1086/655811

Appendix Table 1. Respondent characteristics

Characteristics	No. respondents (%), n = 1715	Effect Size*
Sex		0.27
Male	539 (31)	
Female	1176 (69)	
Age group, y		0.82
18–24	441 (26)	
25–34	558 (33)	
35–44	381 (22)	
45–54	197 (11)	
≥55	138 (8)	
Education attainment		1.14†
Lower secondary or below	58 (3)	
Higher secondary	302 (18)	
Diploma	274 (16)	
Degree or above	1081 (63)	
Living district		0.27
Hong Kong Island	307 (18)	
Kowloon West	128 (7)	
Kowloon East	268 (16)	
New Territories West	471 (27)	
New Territories East	541 (32)	
Employment status		0.57†‡
Employee	1106 (64)	
Employer	62 (4)	
Housekeeper	135 (8)	
Student	285 (17)	
Retired	46 (3)	
Unemployed	81 (5)	
Monthly household income (HKD)		Nil§
≤10,000	104 (6)	
10,001–20,000	277 (16)	
20,001–30,000	297 (17)	
30,001–40,000	233 (14)	
40,001–60,000	290 (17)	
>60,000	257 (15)	
Not disclosed	257 (15)	

*Cohen's w effect size.
†Data for 15 y old or above is extracted from 2016 by-census for comparison.
‡The "unemployed" category is excluded from comparison as it is unavailable from 2016 by-census.
§The "monthly household income" category is excluded from comparison as it is unavailable from 2016 by-census.

Appendix Table 2. Background health conditions and travel history of respondents

Characteristics	No. respondents (%), n = 1715
Self-perceived health status	
Very good / good	1331 (78)
Fair	352 (21)
Very bad / bad	32 (2)
Presence of chronic conditions	
Yes	192 (11)
No	1523 (89)
Medical consultation in the past 14 d*	
Yes	293 (17)
No	1422 (83)
Presence of respiratory symptoms in the past 14 d	
Yes	423 (25)
No	1292 (75)
Leave Hong Kong in the previous month	
Yes†	408 (24)
No	1307 (76)
Regular visitors to the Mainland China	
Yes‡	46 (3)
No	1669 (97)

*Both Chinese and Western medical consultations are included.
†Multiple destinations are allowed. Number of respondents (out of 408) who indicated travel outside Hong Kong in the previous month: outside China (294), China - Guangdong province (96), China - other province (13), Macau (29).
‡Number of respondents (out of 46) who indicated regular visit to the Mainland China: daily (4), weekly (7), monthly (21), quarterly (4), and at most quarterly (10).

Appendix Table 3. Comparison of disease severity

Diseases	Very bad	Bad	Neutral	Not bad	Not bad at all
Emerging infectious disease					
COVID-19	1545 (90)	150 (9)	15 (1)	0 (0)	1 (0)
Existing infectious diseases					
SARS	1551 (91)	133 (8)	21 (1)	2 (0)	4 (0)
2009 pandemic influenza	604 (35)	889 (52)	172 (10)	40 (2)	6 (0)
Seasonal influenza	191 (11)	948 (55)	311 (18)	251 (15)	10 (1)
Noncommunicable diseases					
Diabetes	659 (39)	804 (47)	188 (11)	51 (3)	9 (1)
Cancer	1432 (84)	215 (13)	45 (3)	11 (1)	8 (0)
Heart disease	1123 (66)	502 (29)	66 (4)	17 (1)	3 (0)
Acquired immune deficiency syndrome	1354 (79)	257 (15)	69 (4)	22 (1)	9 (1)

Appendix Table 4. Information wanted by the respondents

Information you want to receive about COVID-19	No. (%), n = 1639
Distribution of cases	1506 (92)
Number of people infected	1497 (91)
Interventions of Hong Kong government	1450 (88)
Preventive measures	1424 (87)
Disease progression	1327 (81)
Symptoms/how to know if one is infected	1310 (80)
Interventions of international organizations	1182 (72)
What to do if infected	1087 (66)
Impact on risk groups	1073 (65)
Risks and consequences	1061 (65)
Interventions of Chinese government	1010 (62)

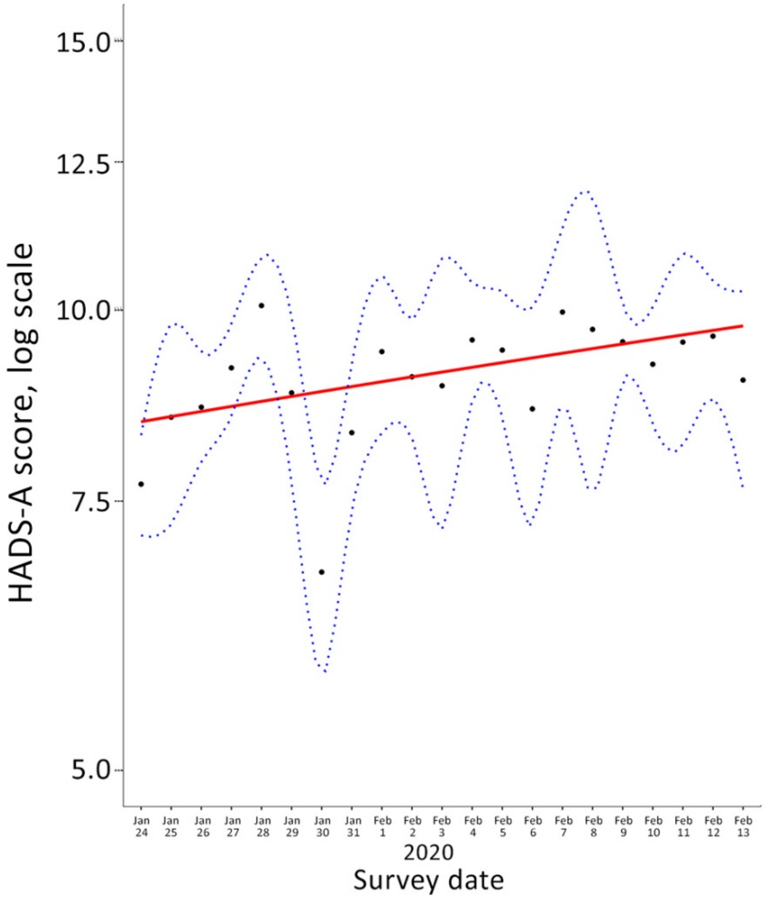

Appendix Figure 1. Time trend of HADS-A score.

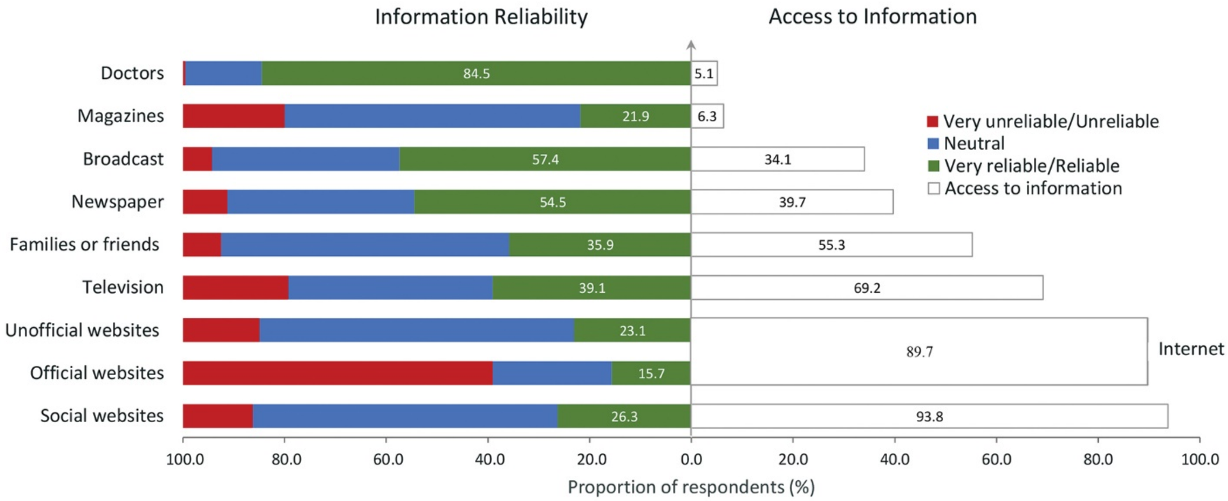

Appendix Figure 2. Information reliability and the access to information of COVID-19.
